# The impact of chronic pain on opioid addiction treatment: a systematic review protocol

**DOI:** 10.1186/s13643-015-0042-2

**Published:** 2015-04-16

**Authors:** Brittany B Dennis, Monica Bawor, James Paul, Michael Varenbut, Jeff Daiter, Carolyn Plater, Guillaume Pare, David C Marsh, Andrew Worster, Dipika Desai, Lehana Thabane, Zainab Samaan

**Affiliations:** 1 Department of Clinical Epidemiology and Biostatistics, McMaster University, 1280 Main Street West, Hamilton, Ontario L8S4L8 Canada; 2 Population Genomics Program, Chanchlani Research Centre, 1280 Main Street West, Hamilton, Ontario L8S4L8 Canada; 3 McMaster Integrative Neuroscience Discovery & Study (MiNDS) Program, McMaster University, 1280 Main Street West, Hamilton, Ontario L8S4L8 Canada; 4 Department of Anesthesia, McMaster University, 1280 Main Street West, Hamilton, Ontario L8S4L8 Canada; 5 Canadian Addiction Treatment Centres, 13291 Yonge St #403, Richmond, Hill, Ontario L4E4L6 Canada; 6 Northern Ontario School of Medicine, 935 Ramsey Lake Rd, Sudbury, Ontario P3E 2C6 Canada; 7 Department of Medicine, Hamilton General Hospital, 237 Barton St East, Hamilton, Ontario L8L 2X2 Canada; 8 Hamilton Health Sciences, Population Health Research Institute, 237 Barton St East, Hamilton, Ontario L8L 2X2 Canada; 9 Departments of Pediatrics and Anesthesia, McMaster University, 1280 Main Street West, Hamilton, Ontario L8S4L8 Canada; 10 St. Joseph’s Healthcare Hamilton, Centre for Evaluation of Medicine, 50 Charlton Avenue East, Hamilton, Ontario L8N 4A6 Canada; 11 Biostatistics Unit, Father Sean O’Sullivan Research Centre, St. Joseph’s Healthcare Hamilton, 50 Charlton Avenue East, Hamilton, Ontario L8N 4A6 Canada; 12 Department of Psychiatry and Behavioral Neurosciences, McMaster University, 100 West 5th Street, Hamilton, Ontario L8N 3K7 Canada; 13 Peter Boris Centre for Addictions Research, 100 West 5th Street, Hamilton, Ontario L8N 3K7 Canada

**Keywords:** Chronic pain, Opioid maintenance, Addiction, Opioid substitution therapies, Opioid dependence, Buprenorphine/naloxone, Methadone, Methadone maintenance therapy, Naltrexone, Systematic review, Network meta-analysis

## Abstract

**Background:**

The consequences of opioid relapse among patients being treated with opioid substitution treatment (OST) are serious and can result in abnormal cardiovascular function, overdose, and mortality. Chronic pain is a major risk factor for opioid relapse within the addiction treatment setting. There exist a number of opioid maintenance therapies including methadone, buprenorphine, naltrexone, and levomethadyl acetate (LAAM), of which the mediating effects of pain on treatment attrition, substance use behavior, and social functioning may differ across therapies. We aim to 1) evaluate the impact of pain on the treatment outcomes of addiction patients being managed with OST and 2) identify the most recently published opioid maintenance treatment guidelines from the United States, Canada, and the UK to determine how the evidence is being translated into clinical practice.

**Methods/design:**

The authors will search Medline, EMBASE, PubMed, PsycINFO, Web of Science, Cochrane Database of Systematic Reviews, ProQuest Dissertations and theses Database, Cochrane Central Register of Controlled Trials (CENTRAL), World Health Organization International Clinical Trials Registry Platform Search Portal, and the National Institutes for Health Clinical Trials Registry. We will search www.guidelines.gov and the National Institute for Care and Excellence (NICE) databases to identify the most recently published OST guidelines. All screening and data extraction will be completed in duplicate. Provided the data are suitable, we will perform a multiple treatment comparison using Bayesian meta-analytic methods to produce summary statistics estimating the effect of chronic pain on all OSTs. Our primary outcome is substance use behavior, which includes opioid and non-opioid substance use. We will also evaluate secondary endpoints such as treatment retention, general physical health, intervention adherence, personal and social functioning, as well as psychiatric symptoms.

**Discussion:**

This review will capture the experience of treatment outcomes for a sub-population of opioid addiction patients and provide an opportunity to distinguish the best quality guidelines for OST. If chronic pain truly does result in negative consequences for opioid addiction patients, it is important we identify which OSTs are most appropriate for chronic pain patients as well as ensure the treatment guidelines incorporate this information.

**Systematic review registration:**

PROSPERO CRD42014014015 http://www.crd.york.ac.uk/PROSPERO/display_record.asp?ID=CRD42014014015#.VS1Qw1wkKGM

## Background

Chronic non-cancer pain is a serious comorbidity impacting the lives of over 95 million people, an estimated 30.7% of the US population [1]. Chronic pain is defined as pain lasting longer than 3 months or past the standard time for tissue to heal [2]. Front-line treatments include the prescription of long-acting opioids, although there is minimal evidence to suggest that opioids provide any long-term relief for chronic pain [3]. Trends in current prescribing practice suggest that the rise in prescription opioid use [4] has been paralleled by a concerning increase in opioid-related deaths, addiction, and medication diversion [5-9]. Opioids are highly liable for misuse, which is evident from the reported incidence of addiction, ranging from 3.2% to 27% among the chronic pain population [10].

While methadone is employed in the management of chronic pain, its most common use is in the treatment of opioid addiction [11], known formally as methadone maintenance treatment (MMT). Under the supervision of addiction specialists, methadone (a synthetic opioid) is prescribed to alleviate the symptoms of withdrawal and prevent relapse [11]. Within the addiction population being treated with methadone, chronic non-cancer pain is the most commonly reported comorbidity, with an estimated prevalence ranging from 37% to 55.3% [12-14].

The intersection between pain management, opioid dependence, and addictive behavior inflates the challenges of treating both addiction and chronic pain. In addition to psychiatric disturbance and inadequate social support, chronic pain is known to be one of the greatest risk factors for opioid relapse within the methadone setting [15,16]. These effects are argued to be the result of opioid-induced hyperalgesia, characterized as a status of heightened nociceptive sensitization caused by opioid exposure [17]. This effect has been demonstrated repeatedly, whereby patients with non-cancer chronic pain taking methadone showed increased hyperalgesic response (assessed by cold presser test but not stimulus) in comparison to their placebo-matched controls [17,18].

The risk for abnormal cardiovascular function [19,20], overdose [21,22], and mortality [21] is highest among patients abusing opioids in combination with MMT. Classifying chronic pain as a risk factor for continued opioid abuse [12,15,16,23] calls to question which addiction treatment is most appropriate for patients with comorbid pain. There exist a number of opioid maintenance therapies including methadone, buprenorphine, naltrexone, and levomethadyl acetate (LAAM), of which the mediating effects of pain on treatment attrition, substance use behavior, and social functioning may differ across therapies.

Is chronic pain an important mediating factor when evaluating patient response to opioid addiction treatment? Which opioid maintenance therapy is best for improving physical, psychiatric, and substance use behavior outcomes in patients with opioid addiction and chronic pain? We aim to evaluate these questions using evidence gathered from all studies evaluating chronic pain in the opioid addiction patient population. The lack of current summary of evidence evaluating the mediating effects of pain suggests that our current effort to combine the evidence will serve to 1) distinguish the best therapy for opioid addiction patients with comorbid pain and 2) enable clinicians to tailor treatments based on an important and highly prevalent risk factor.

### Objectives

We aim to 1) evaluate the impact of comorbid chronic non-cancer pain on all opioid addiction treatment outcomes reported in the literature including treatment retention, illicit substance-use behavior, as well as physical and psychiatric symptoms, 2) determine how different opioid maintenance treatments compare in their effectiveness for patients with comorbid chronic non-cancer pain, 3) provided the data are suitable, combine the evidence from direct and indirect comparisons using network meta-analysis, and 4) identify the most recently published opioid maintenance treatment guidelines from the United States, Canada, and the UK to determine how the evidence is being translated into clinical practice for addiction management.

### Research questions

1.1 Among patients with opioid addiction being treated with (or randomized to) opioid substitution treatment (OST): 1) does chronic non-cancer pain interfere with the effect of OST, and 2) which OST is best for improving treatment response for patients with comorbid chronic non-cancer pain? We will evaluate response across multiple outcome domains including: substance use behavior, physical health, psychiatric symptoms, as well as personal and social functioning.
1.2 Do the most recently published United States, Canadian, and United Kingdom OST clinical practice guidelines capture and properly translate the evidence obtained from the studies evaluated in this review?

## Methods/design

### Systematic review methods

#### Inclusion and exclusion criteria

To be included in this review, the study must evaluate the impact of chronic pain on patient’s response to opioid addiction treatment. The study must have provided a comparison of response to treatment outcomes (for example, continued opioid abuse, general physical health) between patients with and without chronic pain. We also require the studies to have evaluated patients on an OST for opioid addiction. We will not place any restrictions on the types of OST or measurement of chronic pain. All study designs will be accepted into this review, (that is, randomized controlled trials, observational studies, or qualitative studies). No restrictions were placed on socioeconomic, geographic, or ethnic backgrounds of participants for this review.

To be eligible for inclusion, all studies must be primary (original research in patients with pain, no secondary reporting), completed (no interim analyses will be allowed in this review), and performed in a human population.

#### Outcome measures

The primary outcome in this review is illicit opioid use, which can be measured in various ways including urine toxicology screening or self-report. We anticipate many definitions and measurements of opioid use. For example, some studies measure opioid use behavior as the number of days of opioid use in the last month, while others report the mean number of positive opioid urine screens or days until opioid relapse. We will accept any definition or measurement of illicit opioid use, provided the study performs an analysis comparing opioid use behavior based on patients’ chronic pain status. We will also abstract data on all other efficacy end-points including non-opioid substance abuse, general physical health, psychiatric symptoms, personal and social functioning, intervention adherence (for example, treatment retention, dropout rate), resource utilization (for example, hospital admissions) as well as treatment preference. However, short-term outcomes (initial dosing, initial response in a period of <3 weeks, or early detoxification response) will not be evaluated.

#### Data sources and search strategy

We will perform an electronic search using the Medline, EMBASE, PubMed, PsycINFO, Web of Science, Cochrane Database of Systematic Reviews, ProQuest Dissertations and theses Database, Cochrane Central Register of Controlled Trials (CENTRAL), World Health Organization International Clinical Trials Registry Platform Search Portal, and the National Institutes for Health Clinical Trials Registry. In addition, the reference lists of all Cochrane reviews addressing this topic will be reviewed. We will use the Cochrane reviews to validate our own searches of databases and ensure that we have captured the relevant articles in our field. This supplementary search will be applied to Cochrane reviews since they are considered the gold standard in systematic reviews.

We will use a comprehensive search strategy tailored for each database. Please refer to Table 1 for an outline of the search strategy. We consulted a McMaster University Faculty of Health Science librarian as needed throughout the design and investigation phases of the study. The search will be restricted to human studies. Our search will not be restricted to the published literature. We acknowledge that studies in the unpublished literature may not be subject to the same scrutiny as the investigations published in peer-reviewed journals. However, the unpublished literature meeting the inclusion criteria will still be subject to the same rigorous risk of bias assessment as all studies included in this review. To ascertain the gray literature, we will perform a search using the ProQuest Dissertations and theses Database. The title, abstract, and full-text screening will be performed in duplicate by two independent reviewers (Dennis, B and Bawor, M).

**Table 1:** **Electronic search strategy for the identification of relevant studies across multiple databases**

Databases	Search strategies
MEDLINE Search = ______	1. substance related disorders.mp. or Substance-Related Disorders/
	2. opioid related disorders.mp. or Opioid-Related Disorders/
	3. Opioid-Related Disorders/or Methadone/or Analgesics, Opioid/or Heroin Dependence/
	4. 1 or 2 or 3
	5. methadone.mp. or Methadone/
	6. Opiate Substitution Treatment/or Naloxone/ or Buprenorphine/or Opioid-Related Disorders/or Narcotic Antagonists/
	7. buprenorphine.mp. or Buprenorphine/
	8. naltrexone.mp. or Naltrexone/
	9. Substance Abuse Treatment Centers/or Heroin/or Heroin Dependence/or Opioid-Related Disorders/or Randomized Controlled Trials as Topic/or Methadone/
	10. opioid substitution treatment.mp. or Opiate Substitution Treatment/
	11. Buprenorphine/or Analgesics, Opioid/or Opioid-Related Disorders/or Methadone/or Heroin Dependence/
	12. 5 or 6 or 7 or 8 or 9 or 10 or 11 or 12
	13. chronic pain.mp. or Chronic Pain/
	14. 4 and 13 and 4
	15. limit 15 to humans
Web of Science Search = ______	1. Topic = (“methadone” OR “methadone maintenance therapy” OR “naltrexone” OR “suboxone” OR “buprenorphine” OR “heroin assisted treatment”)
	2. Topic = (“opioid dependence” or “addiction”)
	1. Topic = (“chronic pain” OR “pain” OR “opioid induced hyperalgesia”)
	2. 1 AND 2 AND 3
EMBASE = _____	1. methadone treatment/or methadone.mp. or methadone/or methadone plus naloxone/
	2. heroin dependence/or maintenance therapy/or methadone/or opiate addiction/or diamorphine/or methadone treatment/
	3. buprenorphine/or buprenorphine.mp.
	4. naltrexone.mp. or morphine sulfate plus naltrexone/or naltrexone/
	5. opioid substitution treatment.mp. or opiate substitution treatment/
	6. methadone/ or diamorphine/or heroin dependence/
	7. levomethadyl acetate.mp. or levacetylmethadol/
	8. 1 or 2 or 3 or 4 or 5 or 6 or 7
	9. substance related disorder.mp. or addiction/
	10. naltrexone/ or buprenorphine/or opioid addiction.mp. or methadone/
	11. 9 or 10
	12. chronic pain.mp. or chronic pain/
	13. 8 and 11 and 12
	14. limit 13 to human
PsychINFO Search = _____	1. exp Drug Therapy/or exp Methadone Maintenance/or exp Heroin Addiction/
	2. exp Methadone/or exp Naloxone/or exp Drug Therapy/or exp Drug Dependency/or buprenorphine.mp.
	3. naltrexone.mp. or exp Naltrexone/
	4. exp Heroin Addiction/or exp Drug Rehabilitation/or exp Drug Dependency/or exp Clinical Trials/
	5. exp Drug Therapy/or exp Methadone Maintenance/
	6. 1 or 2 or 3 or 4 or 5
	7. exp Drug Abuse/or substance related disorder.mp. or exp Drug Dependency/
	8. substance abuse.mp. or exp Drug Abuse/
	9. 7 or 8
	10. chronic pain.mp. or exp Chronic Pain/
	11. 6 and 9 and 10
Cochrane Library: Cochrane Review and Cochrane Central Register of Controlled Trials = _____	Search title, abstract, keywords:
	1. “methadone” OR “naltrexone” OR “buprenorphine” OR “opioid substitution treatment” OR “levo-methadyl acetate” OR “heroin assisted treatment” OR “heroin substitution treatment”
	2. “substance abuse disorder” OR “opioid abuse” OR “substance-related disorder” OR “opioid addiction”
	3. “chronic Pain” OR “pain” OR “hyperalgesia” OR “neuropathic pain”
Clinical Trials Registry through National Institutes for Health = _____	“methadone” OR “suboxone” OR “Buprenorphine” OR “substitute opioid therapy” OR “naltrexone” OR “heroin assisted treatment” OR “heroin adjustment therapy” AND “opioid addiction” AND “chronic pain”, with additional criteria including: Completed studies, all trials had to be listed as Phase 3, 4
World Health Organization International Clinical Trials Registry Platform Search Portal = _________	“‘opioid addiction’ OR ‘opioid substitution treatment’ OR ‘opioid maintenance treatment’ OR ‘methadone maintenance treatment’” AND “chronic pain”
ProQuest Dissertations and theses Database = _____	“opioid addiction” OR “opioid dependence” AND “pain” OR “Chronic Pain”

#### Selection of studies

Two independent reviewers will screen titles and abstracts and potentially eligible full-text articles using predefined inclusion criteria. Any disagreements or variability between reviewers will be resolved by discussion. If discussion does not lead to a resolution, a third author (Samaan, Z) will be consulted and have the final judgment over the disputed article. We will calculate and report the kappa statistic for each stage (title, abstract, full-text) of screening to display the level of agreement between reviewers.

**Figure 1:**
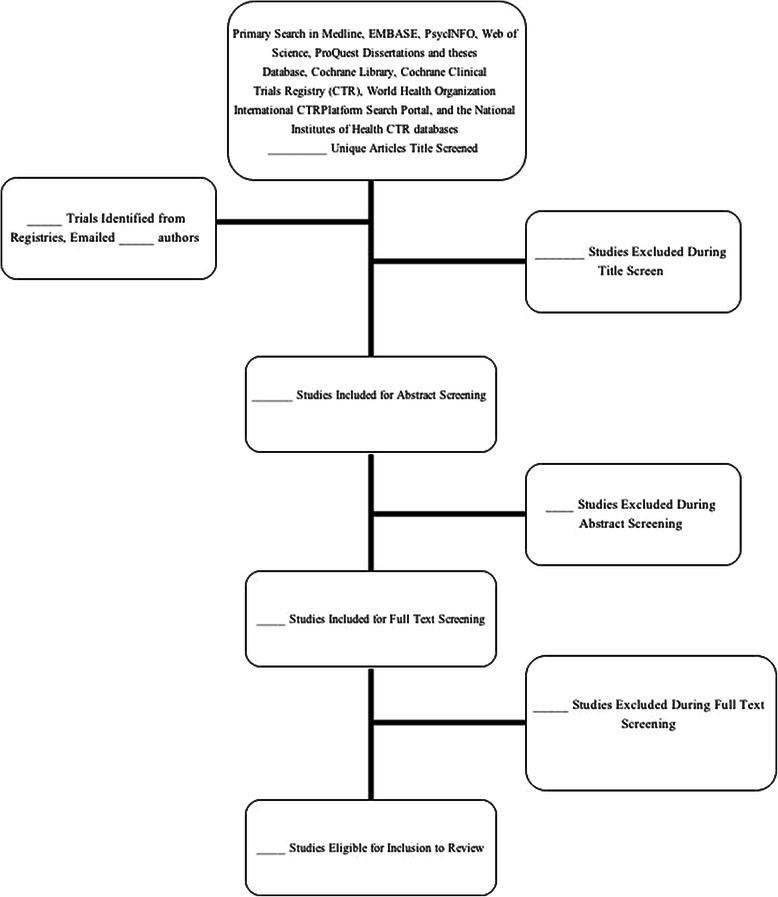
Flow diagram of article screening process.

This review will be reported in accordance with the PRISMA guidelines [24]. The review will include a flow diagram (Figure 1) of the article screening process.

#### Data abstraction

The two authors (BD and MB) will independently extract data from the studies using a pre-established data extraction form (DEF), which is available upon request. All study information will be recorded onto the DEF and later entered onto an electronic Microsoft Excel sheet. The independent reviewers will extract all eligible studies in duplicate. Similar to the methods for disagreement resolution during the title and abstract screening, the independent reviewers will first discuss the disagreements they have during the data abstraction. When discussion does not lead to a resolution, a third reviewer (Samaan, Z) will provide the final decision over the disagreement.

Information extracted during the data abstraction will include author, date of publication, journal of publication, number of study participants, type of population (clinical, incarcerated, pregnant), eligibility criteria, OST(s), OST dose (by chronic pain status), definition of chronic pain, identification of primary outcome, definition of response outcome(s), measurement of chronic pain, measurement of response outcome(s), percentage/number of participants with chronic pain, statistical analysis performed, study findings, overall statistical findings, factors associated with treatment response (if reported), and author’s conclusions.

#### Assessment of methodological quality

Two independent reviewers will assess the methodological quality of the studies in duplicate using a modified Newcastle Ottawa scale for case-control and cohort studies [25], the NIH National Heart, Lung, and Blood Institute: Quality Assessment Tool for Cross-Sectional Studies [26], and the Cochrane Risk of Bias Tool [27] for randomized controlled trials. As mentioned above, any discrepancies between the independent reviewers will first be resolved by discussion; if discussion does not lead to an adequate solution, a third reviewer (Samaan, Z) will be brought in with the responsibility of resolving the dispute.

All summary estimates obtained from meta-analysis will be subject to evaluation using the Grading of Recommendations Assessment, Development and Evaluation (GRADE) guidelines [28]. Provided the data are appropriate, summary statistics derived for direct and indirect estimates using NMA will also be subject to assessment using the GRADE framework [29].

### Statistical analysis methods

The results of this systematic review will be reported in a narrative and where possible, a combined statistical manner. Agreement levels between the independent reviewers will be measured using the kappa statistic. Provided there is little heterogeneity between studies, we plan to conduct a meta-analysis to derive a summary statistic representing the combined statistical result of multiple studies across our primary outcome (illicit opioid use behavior) and secondary efficacy end-points. As described previously [30], the lack of direct comparisons reported in the literature is a common problem when combining the evidence from studies evaluating OSTs. The majority of studies evaluate new therapies in direct comparison to methadone or placebo, leaving us to question the comparative effectiveness compared to other OSTs. To circumvent this problem, we are proposing using network meta-analysis (NMA) to provide the pooled effect estimates of chronic pain mediating effects on the primary outcome (illicit opioid use behavior) for all OSTs.

Research methodologists highly caution against the pooling of studies with fundamentally different designs, [31,32] largely because of imbalanced susceptibility to selection bias non-randomized studies face [31]. Thus, we will combine the results of randomized and non-randomized studies in separate meta-analyses.

#### Direct comparisons

We will perform a meta-analysis to pool results for our primary outcome as well as all secondary efficacy end-points. Findings abstracted from direct comparisons will be pooled together using a random-effect meta-analysis with Knapp-Hartung (KH) estimator [33]. All analyses will be performed using the metafor and rmeta packages in R [34].

Dichotomous outcome(s) will be combined into a pooled odds ratio, where continuous outcomes (for example, mean number of positive opioid urine screens evaluated by chronic pain status) will be pooled using the standardized mean difference. All direct comparisons will be weighted using the inverse of the variance.

Results from studies deemed eligible for inclusion into the meta-analysis will be presented in a forest plot, with the associated 95% confidence intervals presented. We will calculate and report the inconsistency index (*I*^2^) statistics and *P* values as the measure of heterogeneity in the results of the studies and whether the actual observed difference can be attributable to chance alone [35]. We will interpret the *I*^2^ statistic using the thresholds set forth by the Cochrane Collaboration, these include *I*^2^ of 0% to 40% (might not be important), 30% to 60% (moderate heterogeneity), 50% to 90% (substantial heterogeneity), and 75% to 100% (considerable heterogeneity) [31]. The Egger’s test will be used to assess for publication bias.

We anticipate a study’s scoring on methodological quality assessment as well as differences in measurement selection (for example, urine toxicology screening *versus* self-report) to be important factors accounting for heterogeneity between studies. The methodological quality of individual studies will be captured using the Cochrane Risk of Bias tool, Newcastle Ottawa Scale, and the NIH National Heart, Lung, and Blood Institute: Quality Assessment Tool for Observational Cohort and Cross-Sectional Studies. Subgroup analyses will stratify on the basis of the study’s performance on the risk of bias assessment. We will stratify our analyses on the basis of Cochrane risk of bias responses, whereby studies will be characterized has having an overall ‘high risk of bias’ if at least one domain on the Cochrane risk of bias tool is rated as high risk. Thus, results of any study with ≥1 ‘high risk of bias’ rating across domains will be considered at risk for confounding. For observational studies, we will need to address risk of bias according to the appropriate assessment tools, thus we will not be able to use Cochrane risk of bias across all studies. For cohort and case-control studies, any study with zero stars in ≥1 section will be considered high risk of bias based on the Newcastle Ottawa Scale. According to the Newcastle Ottawa Scale, receiving stars indicates a lower risk of bias. The lack of stars in any section indicates the study has not addressed a possible source of confounding. For cross-sectional studies rated with the NIH tool, any study receiving a ‘fair’ or overall ‘poor’ quality rating will be classified as high risk of bias and included for subgroup analysis. We anticipate the studies with improper adjustment for important confounding variables to have high susceptibility for confounded treatment effects.

We will also stratify our meta-analyses based on outcome measurement. A clear example of how measurement can influence the study results is noted with the measurement of opioid use, where some studies use urine toxicology screening to determine concomitant opioid abuse and other studies use self-report. Self-report is susceptible to social desirability bias, where some patients may be reluctant to report continued opioid abuse in an effort to maintain a positive standing with physicians and clinical staff. Thus, quality of measurement can contribute to large difference in the study findings.

Acknowledging the impact of publication status as a potential source of bias, we will perform sensitivity analysis to determine whether a study’s publication status impacts the observed effect estimates. Studies in the gray literature are not subject to the same level of scrutiny as those in peer-reviewed journals. The peer review process leads to the identification of potential sources of confounding and allows authors to re-perform their analyses by properly adjusting for newly identified sources of error. Thus, some of the unpublished literature may present different treatment effects simply due to the lack of external evaluation. We will evaluate this potential concern by performing an additional sensitivity analysis, stratifying our meta-analyses by the articles publication status.

### Combining direct and indirect evidence: the network meta-analysis

Provided the data are suitable for NMA, we propose building a Bayesian hierarchical model using maximum likelihood estimation to derive summary statistics for binary outcomes. This model will introduce a random effect representing the variation in effect estimates resulting from the comparison itself. Any variation in the random effect will be considered ‘inconsistency’ [36]. This method allows for treatment heterogeneity, sampling variability, and inconsistency [36] while also applying maximum likelihood estimation [36].

Due to the fragility of the NMA, we propose selecting the best evidence for inclusion into the model. Thus, only evidence from randomized trials with ≥200 people in the comparison will be selected for inclusion into the NMA model. We set this sample size requirement to adjust for the high susceptibility of type I error in studies evaluating multiple treatment outcomes.

We will use node splitting to identify inconsistency [37,38], a method that identifies loops with large inconsistency. The inconsistency will be taken into consideration during the interpretation of the results. We will also use the deviance information criterion (DIC) to estimate how parsimonious the data are [37].

Findings from the NMA will be presented using probability statements of treatment effects as well as a ranking of these probabilities, which illustrates each interventions probability of ranking first [39]. We will also graphically display the probability ranks using the surface under the cumulative ranking (SUCRA) line [39].

### Methods for evaluating the clinical guidelines

To identify the most recently published North American guidelines on opioid maintenance treatments, we will search www.guidelines.gov. We will search using the terms ‘opioid dependence, opioid addiction, and opioid substitution treatment.’ We will also search the National Institute for Health and Care Excellence (NICE) database to identify the most recently published guidelines used by the National Health Service in the UK. We will use pilot-tested data abstraction forms to extract data on: the recommendations made by each guideline, the strength of the recommendation, the evidence cited by the guideline for each recommendation, whether the guideline developers interpreted any clinical subgroup effects with caution, and whether the guideline discussed the impact of pain on poor treatment response. We will also quantitatively appraise the quality of the guidelines using the Appraisal of Guidelines for Research & Evaluation II (AGREE) Instrument, a validated tool used for guideline assessment [40,41]. We will use this tool to assess the transparency in the development of guideline recommendations for chronic pain subpopulations. However, the use of the AGREE II will be unjustified if no formal recommendations are made for managing this population.

## Discussion

Understanding the impact of comorbid disorders on addiction treatment outcomes is essential for enhancing evidence-based practices within the field of mental health and addiction. This investigation will focus on determining the role that chronic non-cancer pain has on the patient’s experience of opioid addiction treatment. Acknowledging the complexity of comorbid pain management within the addiction treatment setting, we aim to understand the extent to which chronic pain is related to negative health outcomes including functional disability, physical difficulty, and mental health problems such as depression and anxiety in the context of opioid addiction [10]. Determining the influence of chronic pain on response to OST will require a detailed assessment across several different patient important outcomes. This review will capture the experience of treatment for a substantive sub-population of opioid addiction patients. If chronic pain truly does result in negative consequences for opioid addiction patients, it is important that we identify which OST is most appropriate for chronic non-cancer pain patients. We will also identify how current evidence is translated into practice by thoroughly reviewing international guidelines for OST. We aim to address how addiction treatment guidelines propose managing patients with comorbid pain. This objective provides an opportunity to distinguish the best quality guidelines and ultimately identify future areas for improvement.

## Abbreviations

DEF: Data extraction form
EMBASE: Excerpta Medica DataBase
LAAM: Levomethadyl acetate
MMT: Methadone maintenance therapy
NOS: Newcastle-Ottawa Scale
OST: Substitute opioid therapy
PRISMA: Preferred Reporting Items for Systematic reviews and Meta-Analyses
RCT: Randomized controlled trial
WHO: World Health Organization
